# Imaging of the neurological manifestations of dengue: A case series

**DOI:** 10.4102/sajr.v26i1.2528

**Published:** 2022-11-29

**Authors:** Varsha Rangankar, Divyajat Kumar, Rajesh Kuber, Tushar Kalekar

**Affiliations:** 1 Department of Radiodiagnosis, Dr D.Y. Patil Medical College, Hospital and Research Centre, Pune, India

**Keywords:** dengue, haemorrhagic encephalitis, PRES, ADEM, subdural haemorrhage, splenium, RESLES, cytotoxic lesions of the corpus callosum

## Abstract

Dengue fever, the most common arboviral tropical disease, has shown a rapid increase in incidence over the last few decades. Increasing evidence of the various neurological manifestations in dengue has been documented in the literature. Patients positive for dengue on serology and with neurological manifestations were analysed and included in the present case series and brief review. The cases reveal a spectrum of neurological findings in dengue infection and include dengue haemorrhagic encephalitis, acute disseminated encephalomyelitis (ADEM), reversible splenial lesion syndrome (RESLES), intracranial haematoma, and posterior reversible encephalopathy syndrome (PRES), with a focus on the relevant imaging features.

**Contribution:**

The present case series emphasises the importance of understanding the relevant imaging findings and potential aetiopathogenesis of neurological involvement in dengue infected patients in order to make the correct diagnosis for effective treatment and improved outcome.

## Introduction

Dengue fever, with its rapid spread to previously unaffected areas and increasing severity, has become the world’s most common arthropod-borne arboviral illness. The incidence has grown 30-fold over the past 50 years as a result of increased geographic expansion with over 2.5 billion people residing in dengue-endemic nations. The reported estimate is 390 million dengue infections with more than 90 million apparent illnesses each year.^1^ The World Health Organization (WHO) has assigned India to endemicity group A, where dengue infection is a significant health problem, accounting for one-third of the global disease burden, with an estimated caseload of 33 million per year.^1^

The dengue virus was previously considered a non-neurotropic virus with uncommon neurological complications. However, it was later proven to be neurovirulent with documented presence in cerebrospinal fluid (CSF) on the polymerase chain reaction (PCR) test.^2^ The dengue virus is a small single-stranded RNA virus, which belongs to the genus *Flavivirus*, family Flaviviridae. There are four distinct serotypes, designated DENV1 to DENV4 on the basis of their interaction with antibodies in human blood serum. DENV2 and DENV3 are the most implicated in the varied neurologic manifestations of dengue virus infection.^3^ Dengue-associated encephalopathy and encephalitis, dengue-related immune-mediated syndromes, and ophthalmic neurological disorders are some of the examples of the neurological manifestations of dengue virus infection.

The imaging features of some of the interesting and unusual spectra of neurological manifestations of dengue virus infection with possible aetiopathogenesis and differential diagnoses are documented in this case series.

### Case 1

A 25-year-old man presented with a 5-day history of high-grade fever, chills and one episode of vomiting. On admission, he had a single seizure episode, followed by an altered sensorium and deteriorating neurological status. He also had an erythematous macular rash over his body. The patient tested positive for dengue non-structural (NS1) antigen and immunoglobulin M (IgM) dengue antibody, while the test for dengue immunoglobulin G (IgG) antibody was negative. The haematological investigations at the time of presentation revealed a reduced haemoglobin (7 gm/dL) and thrombocytopaenia with a platelet count of 60 000/µl, which reduced to 40 000/µl over the subsequent two days. The platelet count steadily improved thereafter and was 80 000/µl on the sixth day.

**FIGURE 1:**
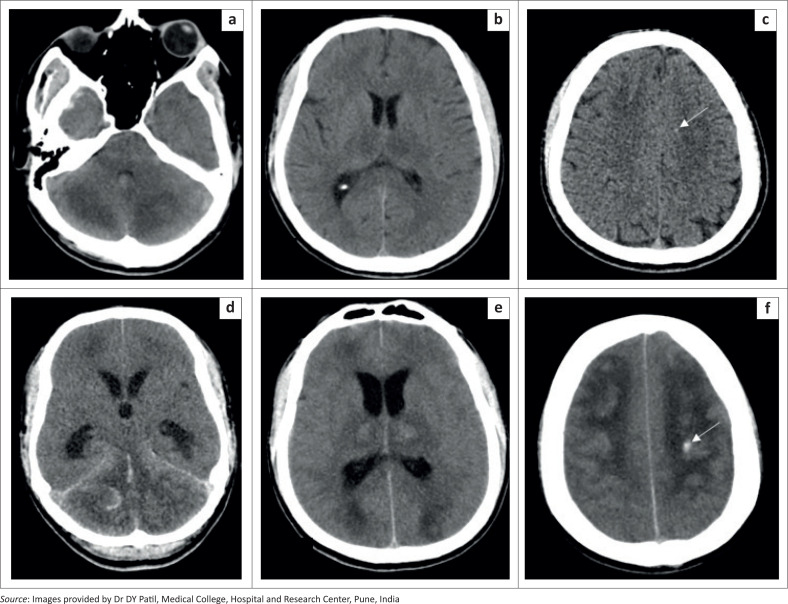
[Case 1]: Axial CT section in a 25-year-old male demonstrating ill-defined symmetric hypodense areas involving the bilateral thalami, cerebellar white matter and bilateral frontal subcortical and deep white matter (a–c). Tiny hyperdense foci were seen within some of these regions (white arrow). A repeat CT study performed a day later revealed an increase in the hypodensities and the haemorrhagic foci with intraventricular extension of the haemorrhage and resultant obstructive hydrocephalus (d–f).

CT imaging of the brain (Figures 1a–c) revealed ill-defined symmetrical bilateral hypodensities in the thalami, cerebellar white matter and bilateral frontal subcortical and deep white matter. Tiny hyperdense haemorrhagic foci were observed within some of these hypodense areas (Figure 1c). Repeat CT Brain imaging (Figures 1d–f), performed a day later due to the patient’s clinical deterioration, revealed an increase in the haemorrhagic foci with intraventricular extension, obstructive hydrocephalus and generalized cerebral oedema.

**FIGURE 2:**
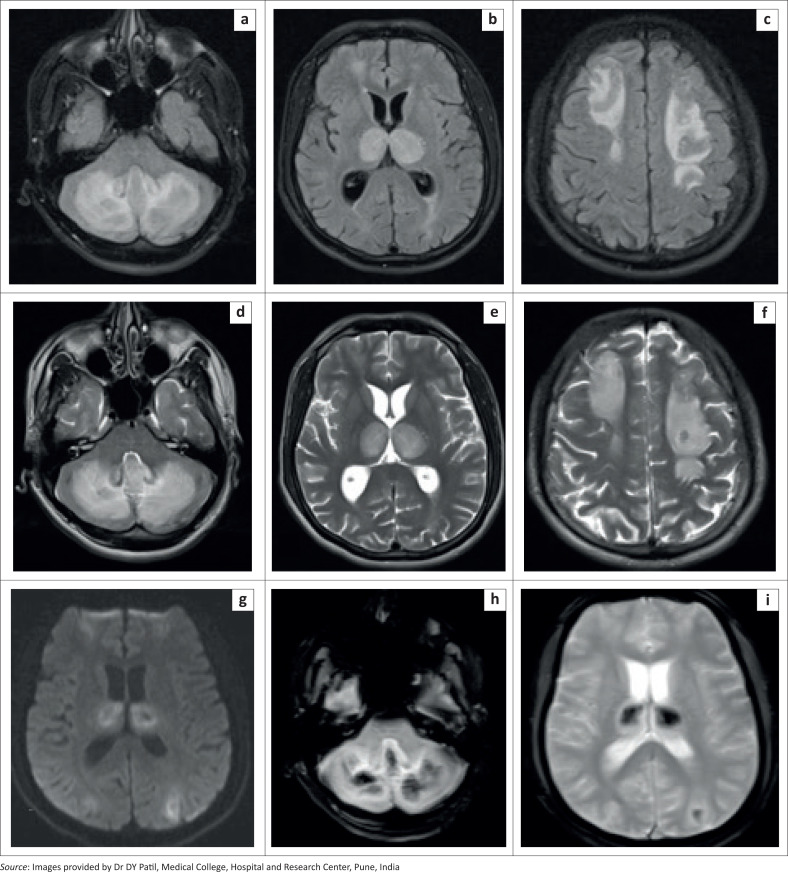
[Case 1]: Brain MRI in a 25-year-old male diagnosed with dengue infection indicates symmetric FLAIR (a-c) and T2 hyperintense areas of altered signal intensity in the vermis, bilateral cerebellar hemispheres, thalami and bifrontal and biparietal white matter (d-f) with patchy diffusion restriction at DWI (g) – ADC not presented. Areas of blooming on GRE images (h, i), representing haemorrhages, are seen.

MRI of the brain (Figures 2a–i) performed on the second day of admission revealed multiple, bilaterally symmetrical, hyperintense areas on fluid attenuated inversion recovery (FLAIR) and T2-weighted images involving the thalami, cerebellum, bilateral frontal and parietal subcortical and deep white matter, with patchy restricted diffusion at diffusion-weighted imaging (DWI) and apparent diffusion coefficient (ADC) (Figure 2g). Multiple internal foci of blooming were seen on gradient recalled echoplanar (GRE) images, representing haemorrhages (Figures 2h and 2i).

A diagnosis of dengue-associated haemorrhagic encephalitis was made based on the clinical presentation, imaging and laboratory findings.

### Case 2

A 10-year-old male presented with a 12-day history of high-grade fever and chills. He also had headaches, retro-orbital pain and occasional episodes of vomiting. He developed an acute onset of right upper and lower limb weakness five days later, followed by weakness of the left upper limb and a decreased level of consciousness. Haematological investigations revealed a platelet count of 80 000/µl with anti-dengue positive IgM and negative IgG status. Dual antigen for malaria and serology markers for Japanese encephalitis and herpes simplex virus (HSV-1) were negative.

**FIGURE 3:**
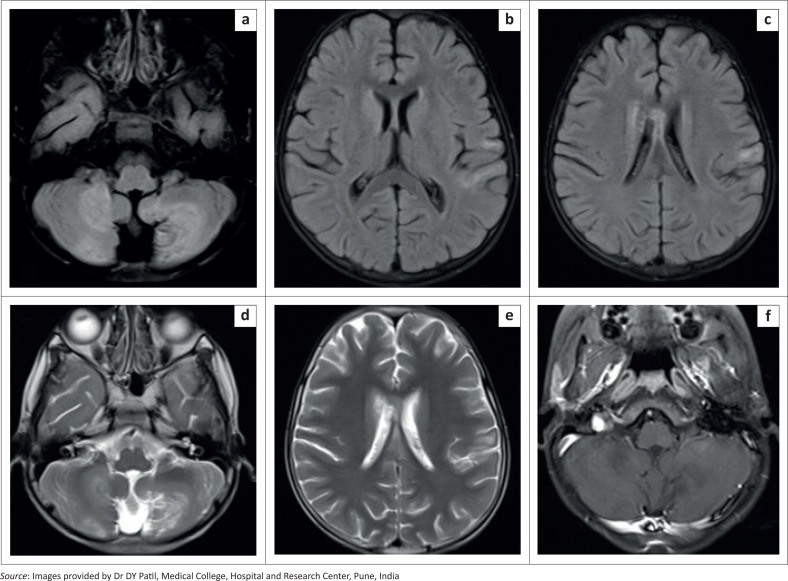
[Case 2]: Brain MRI in a 10-year-old male showing multiple patchy FLAIR (a-c) and T2 hyperintense areas of altered signal intensity in the cerebellar hemispheres, left temporoparietal lobe and body of the corpus callosum, (d, e) with no post-contrast enhancement (f). Subtle FLAIR hyperintense signals were also seen in the inferior part of the medulla (a).

**FIGURE 4:**
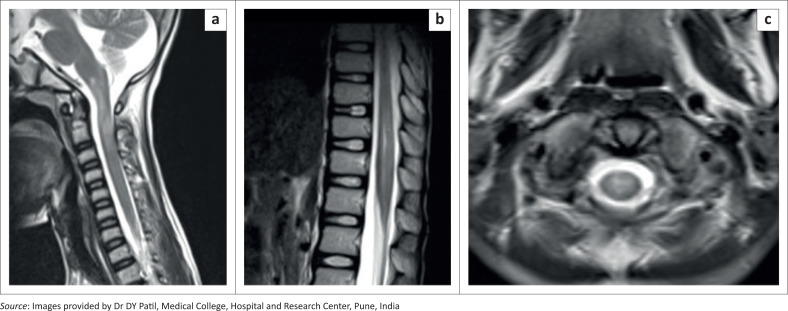
(a–c) [Case 2]: MRI screening of the spine in a 10-year-old male demonstrating T2 hyperintense signal in the inferior part of the medulla, cervicomedullary junction, upper cervical spinal cord, lower dorsal cord and conus medullaris.

Brain MRI revealed multiple patchy hyperintense areas on T2-weighted and FLAIR images in the left temporoparietal lobe and bilateral cerebellar hemispheres, involving the cortex and subcortical white matter, the body of the corpus callosum and the lower medulla (Figures 3a–e). The lesions showed no diffusion restriction and no enhancement on post-contrast T1FS images (Figure 3f). MRI screening of the spine revealed T2 hyperintense signal in the inferior part of the medulla, at the cervicomedullary junction and spinal cord up to the C3 vertebral level as well as in the terminal dorsal cord and conus medullaris from the T11 to the L1 vertebral level (Figure 4a–c).

A diagnosis of acute disseminated encephalomyelitis (ADEM) secondary to dengue infection was made based on the clinical history, laboratory findings and typical MRI findings.

### Case 3

A 20-year-old male presented with a 1-day history of fever, chills and severe vomiting, associated with myalgia, frontal headache and arthralgia. He tested positive for dengue NS1 antigen and was dengue IgM positive on the second day of fever. The patient progressed to a lower level of consciousness over 12 h after admission.

**FIGURE 5:**
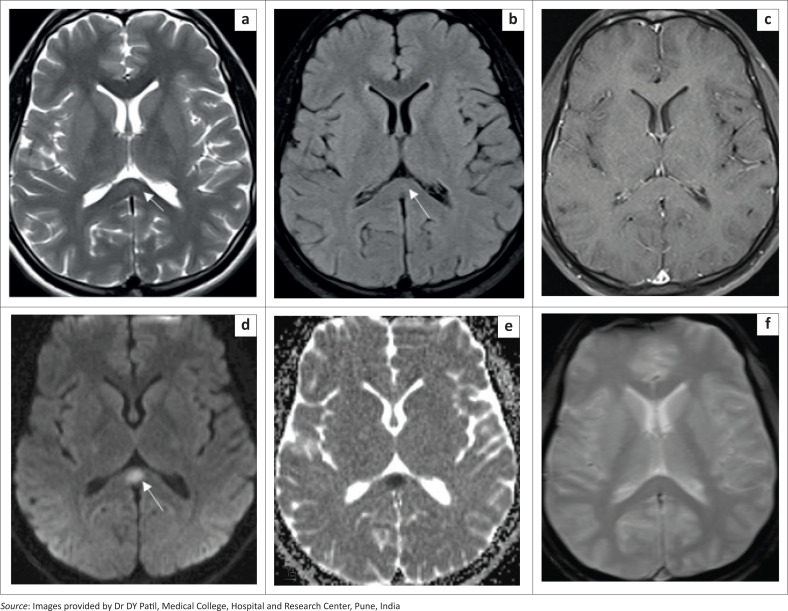
[Case 3]: Brain MRI of a 25-year-old male diagnosed with dengue fever showing a non-enhancing oval shaped T2 (a) and FLAIR (b) hyperintense lesion (white arrow) in the splenium of the corpus callosum. The lesion demonstrated no enhancement (c) and bright signal at DWI (d) and corresponding low values on apparent diffusion coefficient (e) with no blooming on the GRE images (f).

Brain MRI revealed an oval-shaped lesion in the splenium of the corpus callosum, which was hyperintense on T2-weighted and FLAIR images and showed no post-contrast enhancement (Figures 5a–c). The lesion revealed increased signal on diffusion imaging with corresponding low values on ADC and no blooming on GRE images (Figures 5e–f).

The patient was managed conservatively with intravenous fluid, antipyretics and close clinical monitoring. He recovered completely and was released from the hospital on the 14th day of his illness. A follow-up MRI revealed complete resolution of the splenial lesion. A diagnosis of transient lesion of the splenium of corpus callosum, also known as reversible splenial lesion syndrome (RESLES), was made.

### Case 4

A 55-year-old male presented with a 1-day history of left hand and right leg weakness, slurred speech, occipital headache and giddiness. He also had a single episode of convulsions. He reported intermittent high-grade fever with chills five days prior to presentation and was subsequently diagnosed with dengue fever with a platelet count of 75 000/µl. The patient was normotensive with no recent trauma or bleeding from any site.

**FIGURE 6:**
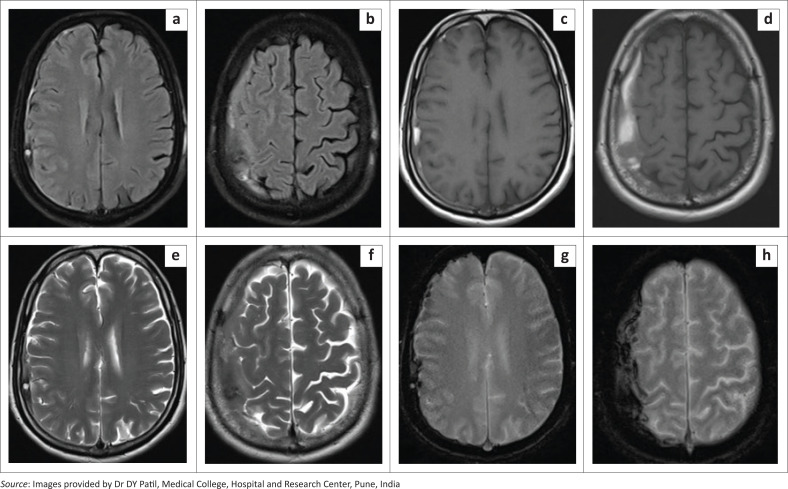
[Case 4]: Brain MRI in a 55-year-old male patient with dengue fever showing a crescent-shaped extra-axial collection overlying the right cerebral convexity, appearing hyperintense on FLAIR (a, b) and T1-weighted (c, d) images and of mixed signal intensity on T2-weighted images (e, f) with blooming on GRE images (g, h), suggestive of an acute to subacute subdural haematoma.

MRI of the brain revealed a well-defined crescent-shaped extra-axial collection overlying the right cerebral convexity which appeared iso to hyperintense on T1-weighted and FLAIR images and of mixed signal intensity on T2-weighted images (Figure 6a–f). The collection demonstrated blooming on GRE images, suggestive of an acute to subacute subdural haematoma (Figures 6g and 6h).

A diagnosis of spontaneous subdural haematoma secondary to dengue virus fever was made.

### Case 5

A 10-year-old female presented to our hospital in a drowsy state with a history of intermittent moderate-grade fever for four days, not associated with chills or rigor. Her blood pressure was 96/66 mm Hg on admission. She tested positive for NS1 antigen and had a platelet count of 35 000/µl. No rashes or bleeding manifestations were present.

**FIGURE 7:**
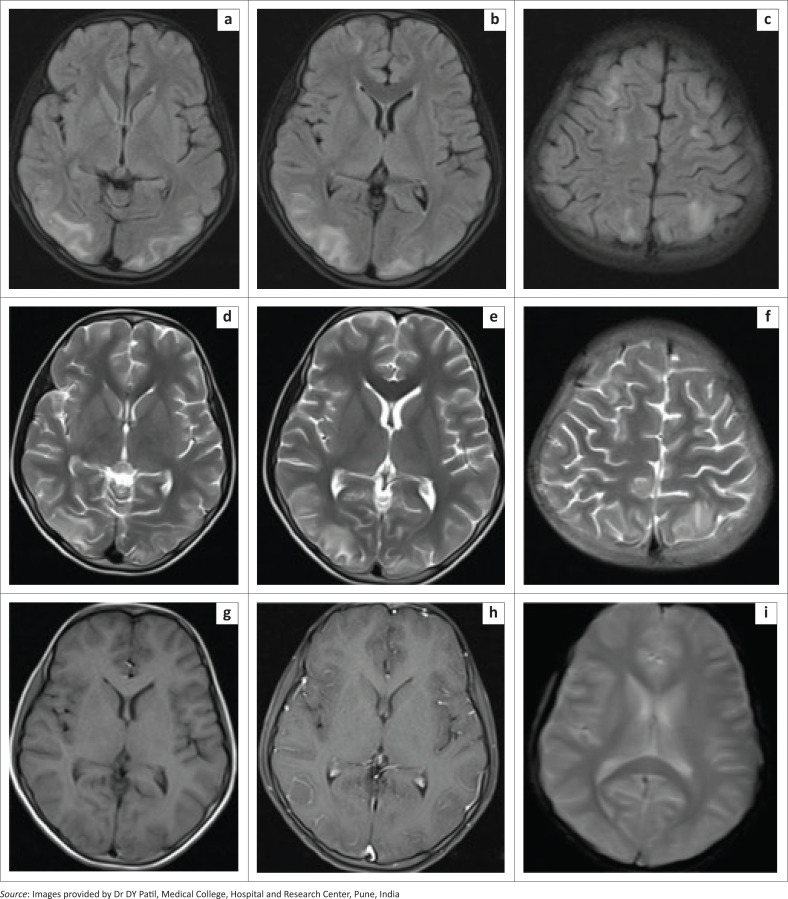
[Case 5]: Brain MRI of a 10-year-old female with NS1 antigen seropositive status showing multiple symmetrical patchy areas of FLAIR (a-c) and T2-weighted (d-f) hyperintensities in the bilateral temporo-occipital regions and to a lesser extent in the frontoparietal regions, involving the cortex and subcortical white matter. These areas showed T1 iso to hypointense signal (g) with no post-contrast enhancement (h) and no blooming on GRE images (i).

MRI of the brain demonstrated multiple, symmetrical, patchy hyperintense areas on T2-weighted and FLAIR images in both temporo-occipital lobes and to a lesser extent in the frontoparietal regions, involving the cortex and subcortical white matter (Figures 7a–f). These areas revealed no diffusion restriction, no blooming on GRE images and no post-contrast enhancement (Figures 7f–i).

Based on the clinical and imaging findings, posterior reversible encephalopathy syndrome (PRES) and dengue encephalitis were considered as possible diagnoses. The patient was managed conservatively and was given dopamine for blood pressure regulation along with methylprednisolone. There was no recorded hypertensive episode throughout the clinical course of the patient in our hospital. The child recovered well and was discharged from the hospital after seven days.

**FIGURE 8:**
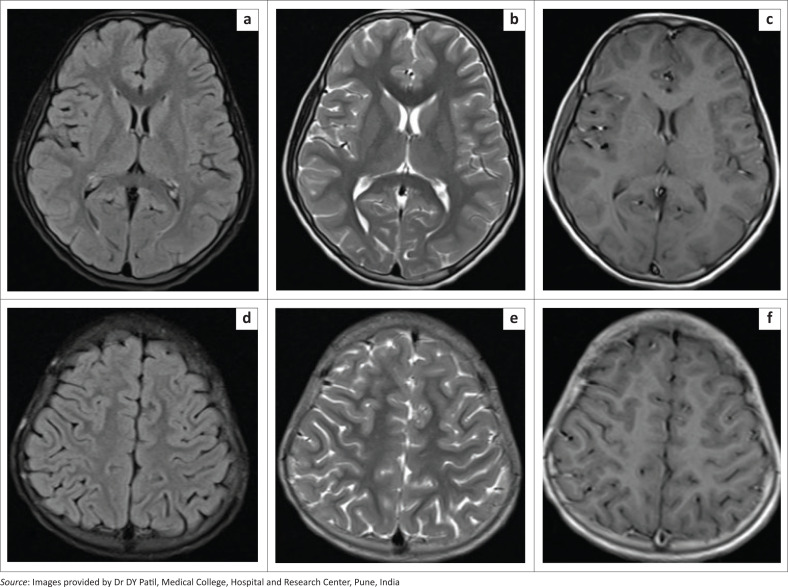
[Case 5]: Repeat brain MRI of a dengue seropositive 10-year-old female diagnosed with posterior reversible encephalopathy syndrome after 4 months showing complete resolution of the symmetrical signal abnormalities in the bilateral temporo-occipital and frontoparietal regions on axial FLAIR (a, d), T2-weighted (b, e) and T1-weighted (c, f) images.

A follow-up brain MRI four months later revealed complete resolution of the signal abnormalities in the bilateral temporo-occipital and frontoparietal regions (Figures 8a–f). In view of the characteristic imaging findings that resolved on follow-up, clinical and laboratory investigations, a final diagnosis of PRES secondary to dengue fever was made.

## Discussion

Neurological involvement in dengue infection is caused by direct virus invasion of the central nervous system (CNS), via an autoimmune reaction and/or metabolic changes.^2^ Although the dengue virus was once considered to have no neurotropism, neurological involvement as evidenced by viral particles found in CSF and disruption of the blood–brain barrier brought on by dengue infection, have all proven otherwise, with frequently reported neurological manifestations.^2,3,4,5,6,7,8^ The incidence of neurological manifestations varies between 2.63% and 40.0%.^2^ The dengue virus may infect both the central and peripheral nervous systems, resulting in varied clinical symptoms.

The various neurological manifestations of the dengue virus are classified based on pathogenetic mechanisms in the recent literature, which also seek to distinguish between dengue-associated CNS and eye involvement, peripheral nervous system involvement and post-dengue immune-mediated or convalescent syndromes.^2,5,6,7,8^ Murthy divided the neurological involvement in dengue infection into three categories in his review of dengue-associated neurological complications.^6^ The first category was due to the neurotropism of the dengue virus, which results in encephalitis, meningitis, myelitis, myositis and rhabdomyolysis. The second category included neurological complications due to systemic effects of dengue infection leading to encephalopathy, haemorrhagic or ischemic stroke, hypokalaemic paralysis and papilloedema. The third category comprised post-infectious immune-mediated complications, which included Guillain-Barré syndrome, ADEM, encephalomyelitis, optic neuritis, neuromyelitis optica and other neuropathies. However, the exact classification might be difficult in reality since these categories overlap and clinical data and adequate investigations may be unavailable.^2^

Central nervous system invasion by the dengue virus and its neurotropic effects have increased the clinical spectrum of encephalitis over the last decade, making it one of the most prevalent neurological presentations. The most common serotypes implicated in creating an encephalitic appearance are DEN-2 and DEN-3.^3^ According to recent data from several researchers, the prevalence of dengue encephalitis ranges between 4.2% and 13.0% of infections of the CNS.^5,7^ The common imaging manifestations of dengue encephalitis include bilateral symmetrical hyperintensities in the thalami, pons and medulla on FLAIR and T2-weighted images, some of which may also show diffusion restriction and petechial haemorrhages.^8^ These show heterogenous or peripheral enhancement after contrast administration. Diffuse cerebral oedema is also often present in these patients. Similar imaging findings and non-specific signs can be seen in patients with Japanese encephalitis, chikungunya encephalitis and herpes encephalitis.^8,9^ Serological testing and the specific anatomical sites of involvement can help to distinguish dengue encephalitis from other infections in challenging instances. Focal lesions on imaging can help to differentiate encephalitis from encephalopathy, which occurs due to multisystem derangement and shows diffuse brain involvement.^6,8^ In the aforementioned case of dengue-associated haemorrhagic encephalitis showing bilateral symmetrical involvement with haemorrhagic foci, the findings are likely secondary to direct neuronal invasion by the dengue virus, as earlier stated by Solomon et al.^5^ Their conclusion was based on the presence of IgM dengue antibody and dengue viruses in the CSF of the dengue patients with encephalitis analysed in their study.

Acute disseminated encephalomyelitis is an immune complex-mediated brain injury that occurs after a viral infection or following immunisation. The proposed pathophysiology is secondary to a transient autoimmune response directed at myelin or other self-antigens, which could be triggered by molecular mimicry and non-specific activation of auto-reactive T-cell clones.^10^ Acute disseminated encephalomyelitis secondary to dengue is relatively rarely encountered with paucity of literature. Three types of lesions may be identified on the brain MRI in patients with ADEM: (1) multifocal white matter lesions with or without basal ganglia involvement, (2) single or multifocal lesions exclusively in the grey matter and (3) localised lesions in the brain stem, basal ganglia or cerebellum.^11^ The presence of hyperintense focal lesions scattered throughout the cortical and subcortical white matter with bilateral cerebellar involvement and associated T2 hyperintensity in the spinal cord in our patient, combined with a history of fever and positive dengue status, lends credence to the diagnosis of ADEM. Japanese encephalitis, herpes simplex encephalitis and ADEM are all typical differential diagnoses for neuroimaging abnormalities in patients with dengue encephalitis. The involvement of the bilateral basal ganglia and thalami in Japanese encephalitis and the bilateral temporal and frontal lobes in herpes simplex encephalitis help to distinguish these pathologies.^11^ Demyelinating lesions on MRI with or without haemorrhagic foci after dengue infection are most likely pathognomonic for ADEM.^10^

Reversible splenial lesion syndrome is a radiological diagnosis marked by a reversible lesion in the splenium of the corpus callosum, caused by both infectious and non-infectious pathologies. Similar imaging findings have been reported in mild encephalitis with reversible splenial lesions (MERS) in cases of contagious aetiologies. Based on the pathophysiology associated with cytotoxic oedema, these transient splenial lesions are sometimes known as cytotoxic lesions of the corpus callosum (CLOCCs).^12^ Although the reason for the preference for the splenium of the corpus callosum is unknown, one theory links it to an absent adrenergic tone and failure of autoregulation in this region.^12,13^ The RESLES or MERS after dengue virus infection was also reported by Fong et al. in which the patient had dengue virus serotype 2 infection with delirium that progressed to ophthalmoplegia.^14^ Complete clinical and imaging resolution of the splenial lesion was also seen in the case reported by Fong et al., which is consistent with the good prognosis associated with this unusual clinical-radiological entity.

Non-traumatic intracranial haemorrhage (ICH) without any other cerebral intracranial complication can be seen in dengue haemorrhagic fever (DHF) and dengue shock syndrome (DSS). Dengue-related ICH can be localised or widespread, typically affecting the cerebrum, ventricles, and less frequently, the cerebellum. Subarachnoid, extradural and subdural haemorrhages (SDH) have also been reported with dengue infection.^15^ Plasma leakage and bleeding are the most common pathophysiological anomalies in DHF and DSS. Capillary fragility and thrombocytopenia lead to bleeding. The majority of people who develop DHF or DSS and have ICH have previously been infected with one or more dengue serotypes.^15^ However, ICHs are still rare compared with frequent dengue fever haemorrhagic symptoms such as haematemesis, melena or epistaxis.^16,17^ Preoperative and postoperative platelet transfusion may be required in patients with ICH requiring operative management.

Posterior reversible encephalopathy syndrome (PRES) is a neurotoxic state that presents with a myriad of features such as seizures, encephalopathy, visual disturbances and headache, typically associated with blood pressure fluctuations or metabolic derangement.^18^ Posterior reversible encephalopathy syndrome can also occur as a complication of various infections or sepsis in normotensive patients.^19^ MRI classically shows vasogenic oedema, typically affecting the subcortical white matter of the bilateral parieto-occipital regions, and less frequently the frontal or temporal lobes and watershed regions, with or without haemorrhagic foci.^18,19^ Posterior reversible encephalopathy syndrome is a rare neurological complication of dengue with a few reported cases. Sawant et al. postulated fluid overload and hypertension due to fluid resuscitation of the hypotensive shock in dengue along with inflammatory cytokine response as likely causes of PRES.^20^ Because the NS1 antigen has a proclivity to act directly on endothelial cells, it causes the release of cytokines and chemokines, which disrupt the integrity of endothelial cell monolayers, resulting in vascular permeability syndrome, which has been proposed as the primary mechanism of PRES in dengue.^18,19^ This theory provides the most likely explanation of PRES in our patient who was in dengue shock. It is critical to distinguish PRES from dengue encephalitis and ADEM because patients with PRES recover well with supportive care.^19,20^

As a word of caution, the characteristic imaging findings described in the various entities in the present case series are not directly pathognomic of dengue. However, dengue should be considered as a strong differential diagnosis in such cases in the appropriate setting of endemicity, clinical presentation and supportive laboratory findings.

## Conclusion

Dengue fever is a major public health issue around the world, particularly in endemic countries such as India. Dengue virus has been shown to be a neurotropic virus with a variety of neurological manifestations. Neuroimaging, particularly MRI, is an essential tool for the early assessment and evaluation of dengue fever neurological manifestations. All the neuroimaging entities described in this report should have dengue as one of the differential diagnoses in the appropriate clinical setting and with supporting laboratory findings. Knowledge of the various dengue infection-related neurological complications and their imaging features can aid in the identification of underlying pathological processes and making the correct diagnosis in an appropriate clinical setting, allowing for proper management and improved prognosis.
